# Translation and adaptation of the sleep apnea quality of life index (SAQLI) to Brazilian Portuguese

**DOI:** 10.1590/0004-282X-ANP-2021-0275

**Published:** 2022-08-08

**Authors:** Amélia Paula Fávero Perrone, Vanessa Ruotolo Ferreira, Lucila Fernandes do Prado, Gilmar Fernandes do Prado, Marco Antonio Machado, Luciane Bizari Coin de Carvalho

**Affiliations:** 1 Universidade Federal de São Paulo, Escola Paulista de Medicina, Departamento de Neurologia, São Paulo SP, Brazil.; 2 Hospital São Paulo, Laboratório de Sono, São Paulo SP, Brazil.; 3 Laboratório de Pesquisa Neuro-Sono, São Paulo SP, Brazil.

**Keywords:** Quality of Life, Sleep Apnea, Obstructive, Translating, Qualidade de Vida, Apneia Obstrutiva do Sono, Tradução

## Abstract

**Background:**

Obstructive sleep apnea syndrome (OSAS) is characterized by episodes of upper airway obstruction during sleep, with a risk of cardiovascular and cerebrovascular diseases. There is no tool in Brazil to measure the impact of treatment on patients with OSAS.

**Objective:**

To translate and culturally adapt the Sleep Apnea Quality of Life Index (SAQLI) into Brazilian Portuguese.

**Methods:**

The translation and cultural adaptation were carried out in five steps: translation, synthesis of the translations, back translation, review committee and pretesting.

**Results:**

A version of a culturally compatible SAQLI was constructed after lexical changes, along with changes to the sentence structures, visual format, instructions and cards. The essence of the questionnaire and its social, emotional, and disease impact in treatment measures was maintained, with 80% understanding.

**Conclusions:**

The questionnaire was translated and adapted culturally to Brazilian Portuguese, and presented good comprehension in the study population.

## RESUMO

**Antecedentes:**

A síndrome da apneia obstrutiva do sono (SAOS) é caracterizada por episódios de obstrução da via aérea superior durante o sono, com risco para doenças cardiovasculares e cerebrovasculares. Não há ferramenta no Brasil para medir o impacto do tratamento em pacientes com SAOS.

**Objetivo:**

Traduzir e adaptar culturalmente o Índice de Qualidade de Vida em Apneia do Sono (SAQLI) para o português brasileiro.

**Métodos:**

A tradução e adaptação cultural foram realizadas em cinco etapas: tradução, síntese das traduções, retrotradução, comitê de revisão e pré-teste.

**Resultados:**

Uma versão de SAQLI culturalmente compatível foi construída após mudanças lexicais, bem como mudanças nas estruturas das frases, formato visual, instruções e cartões, mantendo a essência do questionário e seu impacto social, emocional e da doença nas medidas de tratamento, com 80% de compreensão.

**Conclusões:**

O questionário foi traduzido e adaptado culturalmente para o português brasileiro apresentando bom índice de compreensão na população estudada.

## INTRODUCTION

Obstructive sleep apnea syndrome (OSAS) is a disorder characterized by recurrent episodes of upper airway (UA) obstruction during sleep. It is a multifactorial disease and occurs due to thickening of the UA structures and increased collapsibility of the pharynx^1^.

 A study carried out in the city of São Paulo, Brazil, using polysomnography, identified that the prevalence of OSAS was 32.8%. This may have serious consequences for long-term health, such as: cardiovascular disease, systemic arterial hypertension^2^ ^,^ ^3^, stroke, sexual impotence, cognitive deficit, poorer quality of life and sleep^3^ ^,^ ^4^, decreased work activity and automobile accidents^5^.

 Several instruments have been used^6^ ^,^ ^7^ to assess the quality of life and sleep^6^ among patients undergoing treatment for OSAS^8^ ^,^ ^9^. However, the Sleep Apnea Quality of Life Index (SAQLI) is the first instrument to specifically measure the quality of life of patients with OSAS, before and after treatment. It enables assessment of the impact of treatment on patients' lives, including the negative aspects of using CPAP, intraoral appliances and surgical treatment^10^. 

In Brazil, there is a lack of translated and formally adapted quality-of-life assessment questionnaires to aid healthcare professionals in decision-making and clinical follow-up. Most patients give up on treatment^11^ because of discomfort. The SAQLI has been useful for detecting impairments of quality of life, compared with other questionnaires^12^.

The purpose of this study was to carry out the translation and cultural adaptation of the SAQLI into Brazilian Portuguese.

## METHODS

### Participants

Thirty patients of both genders and 18 to 77 years of age participated in this study. They were recruited from our sleep disorders clinic. The study was conducted from August to November 2015.

### Instrument

After receiving authorization from the original authors of the SAQLI for its translation and cultural adaptation, we started this process in accordance with a standardized guide for this purpose^10^.

The SAQLI is an instrument with four domains that aims to assess the quality of life of patients with OSAS after undergoing some type of treatment5: CPAP, surgery, intraoral device or weight loss. These four domains are as follows: A: daily functioning (11 items); B: social interactions (13 items); C: emotional functioning (11 items); and D: symptoms (5 items). For domains A to D, each item is scored from 1 to 7, with 1 corresponding to the least impairment and 7 to the greatest impairment. An additional domain, E, titled treatment-related symptoms, was added to be used post-treatment and domain F measures the impact of treatment-related symptoms. The translation and cultural adaptation were carried out in five stages: translation, synthesis of translations, back-translation, review committee and pre-test. 

### Procedure

The translation was done by two independent evaluators who were fluent in English and Portuguese. A conceptual rather than a literal translation was emphasized. Next, a meeting was held between the main author, two psychologists and a neurologist specializing in sleep, in order to compare the two translations and reach a consensual synthesis from them: this then constituted version A of the translation. Version A was then back-translated by two other independent evaluators who were native English speakers and fluent in Portuguese^13^.

This was followed by another meeting, of a review committee for synthesis of versions, at which discrepancies between the original English-language instrument, the Portuguese translation and the back translation were documented and analyzed. This process ultimately led to reaching a consensus translation (version B) that would be applied to the population. The instructions for the questionnaire and its items were adapted considering semantic, conceptual, cultural and idiomatic equivalences. Two pretests were then conducted, one in the period from November 2014 to June 2015, among 10 adults, and the other in the period from August to November 2015, among 20 adults.

### Ethical considerations

This study was approved by the Ethics Committee of UNIFESP, São Paulo, Brazil, and all participants signed a consent form.

## RESULTS

Version B of the translated instrument underwent adaptations relating to semantic equivalences (four changes), cultural equivalences (18) and conceptual equivalences (37), but none with regard to idiomatic equivalences. These modifications are described below.

### Semantic equivalence

In item A (daily functions), “I. Most important daily activity. Regarding the execution of your most important daily activities (for example, work, school, childcare, housework, etc.) during the last four weeks”, was modified to: “I. Choose a daily activity that is most important to you (for example, work, school, childcare, housekeeping, etc). Tell us how things have been in the last four weeks regarding this activity”.

 Questions 1, 2, and 3 of item I underwent alterations to the verb tense of the sentence, with use of a simpler and more usual verb structure, to facilitate understanding of the question. In question 3 of item III, the word “conflito” was omitted and only the word “discussões” was used, which is more colloquial in Brazilian Portuguese^14^.

### Conceptual equivalence

In questions 2, 3, and 4 of item I, the expressions “com que frequência” and “quanto tempo” were changed to “por quanto tempo” in order to give a sense of continuity. Some words in the instructions and questions of items 1, 2 and 4 of item II were suppressed, in order to make the sentence more objective and adapt it to the interviewees' understanding. In question 3 of item III, the word "brigar" was changed to "lutar", to achieve the meaning that the person strives to stay awake. In question 2, the word "quarto" was replaced by the word “cômodo” because the home may not always have a bedroom available. In question 6, “quão culpado você se sentiu” was modified to “quanto você se sentiu culpado” because it is the most used and best understood way to ask that question in Brazilian Portuguese. In sentences 7, 8, and 9 of item III B, the phrase “com que frequência” was replaced by “quantas vezes”. In question 11 of item III B, a structural change was made, from "quanto você teve de problema por não estar envolvido…..” to "quanto você teve de problema por não participar”, because this way of asking the question allows respondents to demonstrate whether or not they were willing to participate in activities with family members.

In item C, in the instructions, the sentence was simplified to facilitate understanding. In the questions relating to this item (1, 2, 3, 4, 5, 6, 7, 8 and 9), the structure "com que frequência" was changed "quanto tempo" because this is a phrase more commonly used by patients. The instructions of item D were modified in order to make them more direct. In question 7, the word “frequentemente” was removed in order to simplify the question; while in question 19, the words “relutância” and “incapacidade” were suppressed. In the instructions for item E, the word "circule" was replaced by "indique", and in question 18, the word “autoconsciência” was changed to “sensação de aumento da percepção do rosto/boca”. The instructions for item F were simplified by using fewer words and making them more objective.

### Cultural equivalence

In the initial instructions of the questionnaire, the word “impacto” was replaced by “como .... afeta” in order to reach a more direct explanation and demonstrating how OSAS influences the lives of the respondents. In question 3 of item II, the structure of the question was changed from “Quanta dificuldade você teve relacionada à sua capacidade para exercitar e/ou fazer atividades que você não considera relaxantes” to “Quanto você não se sentiu capaz para.... (atividade que escolher)”, thereby making the question more objective and colloquial. In order to ascertain whether the ability to do leisure activities is still preserved, the instructions for item II B were modified to allow respondents to think about the activities that would be asked about next. In questions 1, 2, 3, 4, 5, 10, 12 and 13 of item III B and questions 10 and 11 of item C, the structure “Quão apreensivo...” was changed to “Quanto você ficou chateado... .” because these words are more appropriate to the colloquial vocabulary of Brazilian Portuguese.

In question 2, the word " fadiga " was changed to " cansaço ", because in the technical test the patients did not understand the word " fadiga ". In question 5, the words " adormecer se não for estimulado " were changed to " adormecer quando não está fazendo nenhuma atividade”, both from item D. In the instructions for item E, the word “circule” was changed to “indique”, so as to have a simpler way of informing the alternative. The format of the instructions in item A was also changed, as reported in item I of the Results.

 The application of the questionnaire was maintained, but the scoring of the cards was simplified. In the original questionnaire, the points go from 1 to 7; however, the sort order is descending. The modification made was that the naming of the classification, i.e. the points from 1 to 7, was kept, but there was no correlation with the naming of any of the points except for points #1 and #7. There was also an inversion regarding the classification. In the original SAQLI in English, the score is presented in descending order; in our study, after application of the pretest and the consensus meeting, it was decided that an ascending order of classification would be adopted, because this meant that the understanding of the response measurements became more direct and did not confuse the interviewees.

 Version B was pretested but required changes. Therefore, version C was applied in the technical test, and this version was shown to have an 80% understanding rate in the population to which it was applied. Thus, no further modifications were deemed necessary.

## DISCUSSION

In this process of translation and cultural adaptation to Brazilian Portuguese, changes were made that were related to cultural, semantic, conceptual and idiomatic equivalences^13^ ^-^ ^15^. The SAQLI has now undergone validation, translation and cultural adaptation in several western and eastern countries. Several of these studies have demonstrated the internal validity, reliability and sensitivity of this instrument, through measurements that identify impairments^16^ ^,^ ^17^ in different areas relating to quality of life, including OSAS specifically^18^ ^-^ ^22^.

 Validation, translation and cultural adaptation studies have shown that the translation from English into the language in which the questionnaire will be used should not be literal but should “convey the spirit of the items of the questionnaire in different languages and cultures”^23^ ^,^ ^24^.

 In addition, the adaptations should encompass different social classes, with different cultural and socioeconomic levels, as seen throughout Brazil, so as also to include functionally illiterate individuals^14^. 

A few semantic changes were made in the present study, and the results from this adaptation give the instrument the possibility of being sensitive to the target population. The first measure that was used to achieve conceptual equivalence was to consult the lexical references of Canadian and Brazilian cultures, which provided the conditions for changes, such as in the expression "com que frequência" and "quanto tempo", which were modified to "por quanto tempo” in order to give a sense of continuity; and likewise, from “com que frequência” to “quantas vezes”. In addition, the phrase “quanto você teve de problema por não estar envolvido...” was changed to “quanto você teve de problema por não participar”.

The word “impacto” was replaced by “como….afeta” and “quão apreensivo…” was replaced by “quanto você ficou chateado…”, in order to achieve cultural equivalences. This change was needed because these terms did not maintain a correlation with the cultural context within which they were being applied^14^. 

Although the study sample encompassed a heterogeneous population in socioeconomic and cultural terms, it was not very large in terms of quantity.

 In conclusion, the SAQLI was translated and culturally adapted to Brazilian Portuguese from the original in English and was shown to have a good comprehension index in the population studied. 
